# Simultaneous Occurrence of Breast Cancer Metastases and Cervical Cancer Metastases in the Same Lymph Node

**DOI:** 10.1155/crog/8810943

**Published:** 2026-09-21

**Authors:** Leonie-Sophia Pösch, Theodora Todorovic, Daniela Hagen, Gesine Meili

**Affiliations:** ^1^ Obstetrics and Gynaecology Kantonsspital Winterthur, Winterthur, Switzerland

## Abstract

**Background and Aims:**

The simultaneous occurrence of different cancer types in the same individual is an uncommon event. We present a case of synchronous metastases of breast cancer and cervical cancer in the same lymph node in a patient with a history of cervical cancer with newly diagnosed breast cancer.

**Method:**

This article is a descriptive case report of a single patient treated at our institution. No statistical analysis was performed.

**Results:**

Following the recommendation of our interdisciplinary tumor conference, we began treatment with neoadjuvant chemotherapy in reverse order, with four cycles of carboplatin/Taxol (corresponding to first‐line chemotherapy for cervical cancer), followed by four cycles of EC (epirubicin and cyclophosphamide) (corresponding to first‐line chemotherapy for breast cancer), mammoplasty, and adjuvant radiotherapy, including internal mammary and supraclavicular node irradiation. By adjusting therapy regimens, it was possible to achieve complete remission of both the primary and secondary tumors.

**Conclusion:**

The occurrence of synchronous cervical and breast cancer is extremely rare, with only a few cases reported in the literature. A definitive correlation between the two cancers cannot currently be established due to limited cases. Because of this rarity, treatment planning is particularly complex and requires an individualized, multidisciplinary approach. A combination of systemic therapy, radiotherapy, and surgery may achieve complete remission in selected patients, as we saw in our case. Mortality rates remain unclear due to the limited number of reported cases.

## 1. Introduction

The occurrence of different cancer types in the same individual is sometimes observed, especially for cancers with related etiological factors such as breast cancer with ovarian or endometrial cancer [1]. The synchronous occurrence of cervical cancer with breast cancer, however, is a rare event and brings diagnostic and therapeutic challenges [2, 3]. In this case, we present a patient with synchronous cervical and breast cancer metastases in one and the same lymph node.

## 2. Case Presentation

A 48‐year‐old woman initially presented with hypermenorrhea and HSIL cytology in 2017. Comorbidities were morbid obesity and high blood pressure. There was no family history of breast cancer or cervical cancer. Portio biopsy showed CIN III with microinvasion, pT1a. Hysteroscopy with fractional curettage could exclude endometrial pathology. A further surgical procedure with conization showed intraoperatively a greater extent than expected. The stage was determined as cT2a (FIGO IIa). A further staging with PET‐CT and pelvic MRI confirmed this. Following the recommendation of our interdisciplinary tumor conference, we started a radiochemotherapy (5x cisplatin) with a subsequent brachytherapy.

After initially successful treatment, regular follow‐up visits took place every 3–6 months. In 2021, screening mammography showed a DCIS of 8 cm with suspected microinvasion. Vacuum biopsy confirmed the diagnosis of breast cancer, NST, pT1a (microinvasion < 1 mm), cN0, and cM0.

### 2.1. Investigations

**Figure 1:**
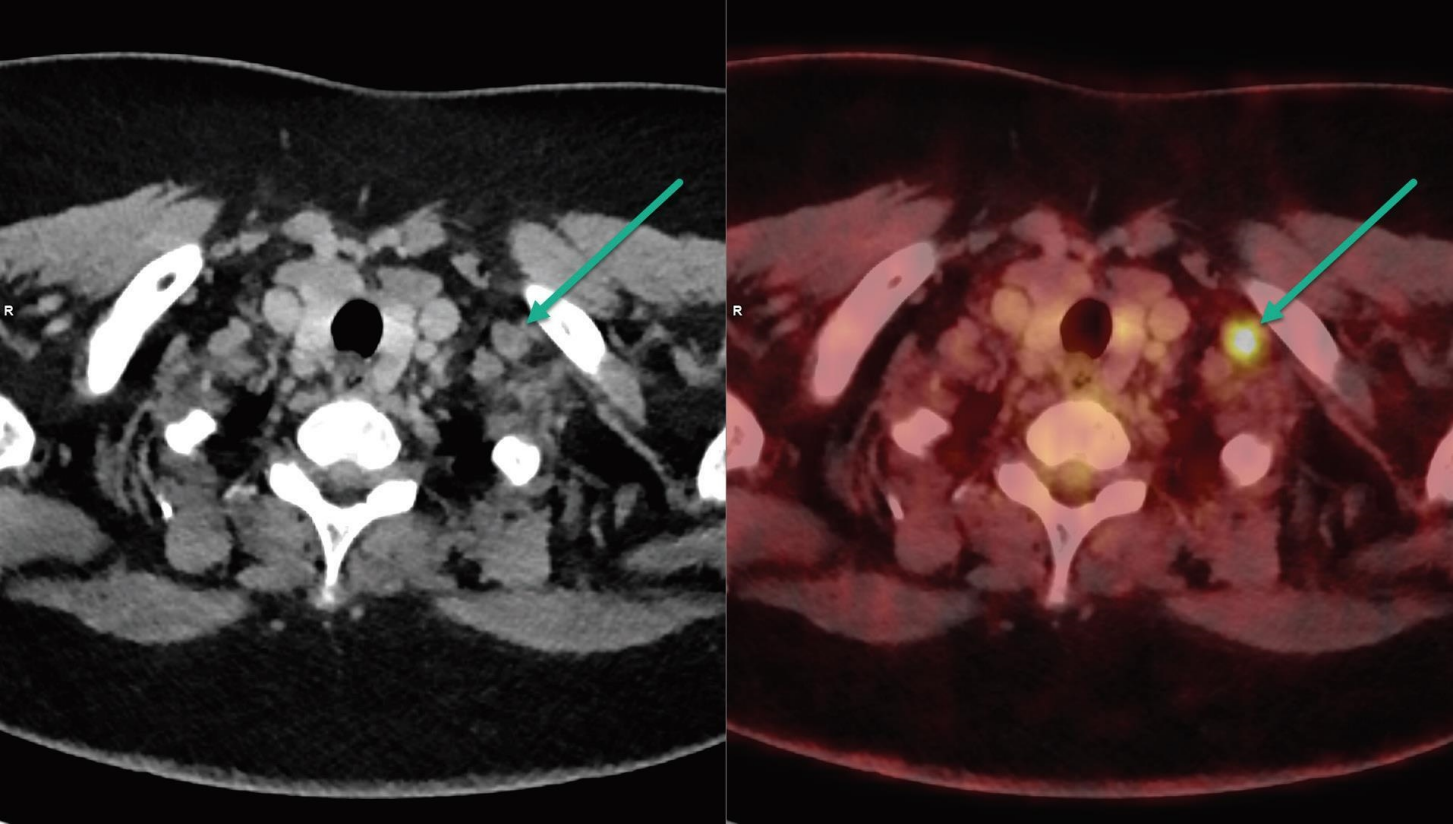
Axial CT and fused 18F‐FDG PET/CT images showing intense FDG uptake in the enlarged left supraclavicular lymph node.

**Figure 2:**
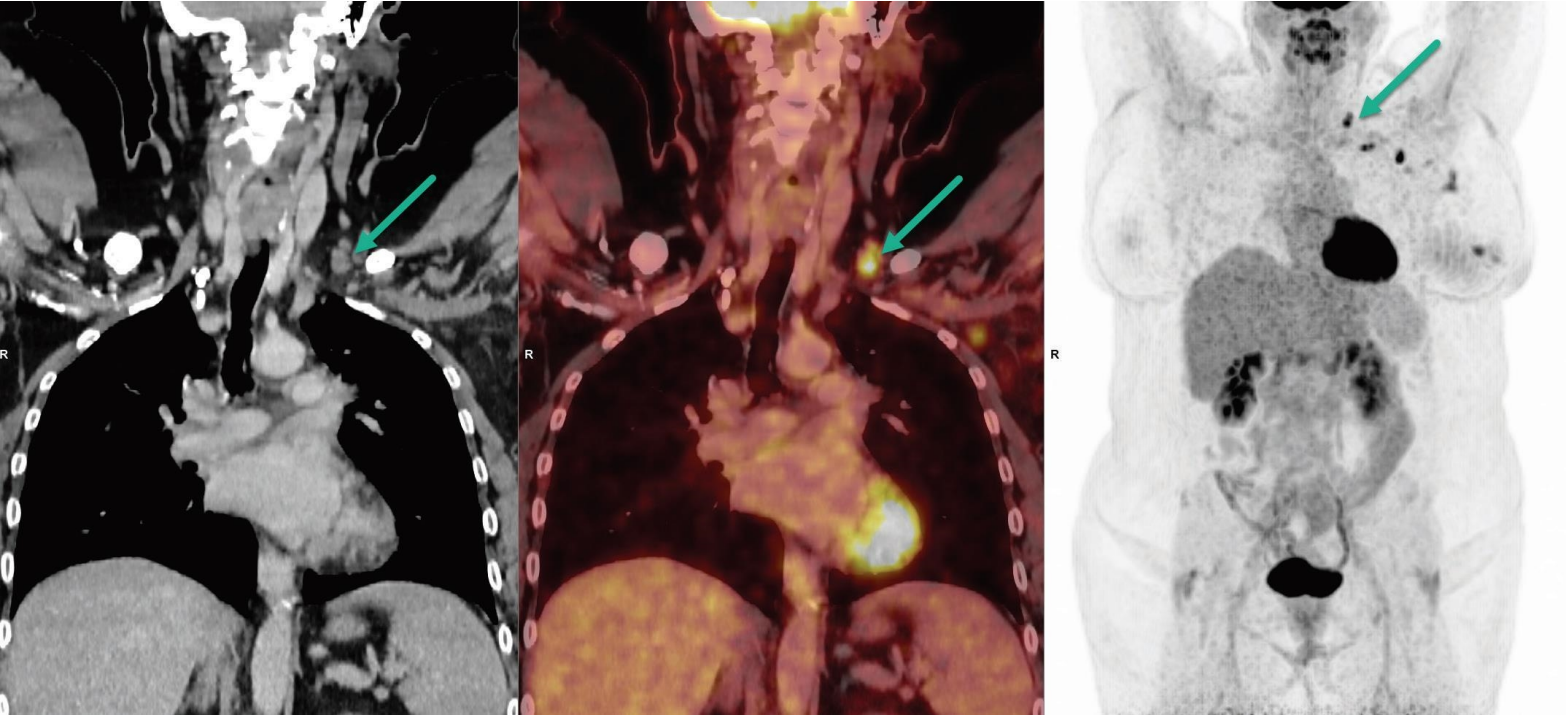
Coronal CT and fused 18F‐FDG PET/CT images and maximum intensity projection coronal images revealing increased FDG uptake in the supraclavicular lymph node metastasis. In addition, increased tracer uptake in the carcinoma in the left breast and the axillary lymph node metastases.

**Figure 3:**
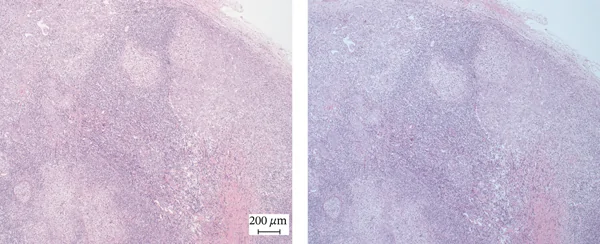
Histopathological image of the left supraclavicular enlarged lymph node showing extensive tumor infiltration. H&E staining.

**Figure 4:**
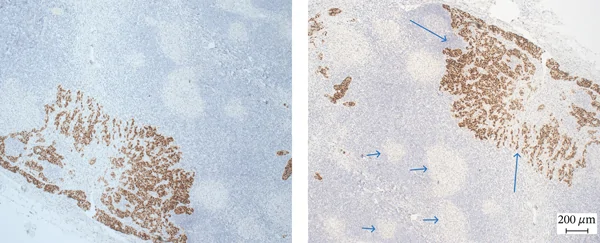
Left supraclavicular lymph node. Immunohistochemistry for the estrogen receptor protein. The staining shows an inverse pattern. Positive staining (long arrows), in contrast to the negative staining (short arrows). An inverse staining pattern was also observed for GATA3, p16, and p40 (not shown here).

Clinical examination proved difficult due to pronounced macromastia; the tumor could not be palpated. Ultrasound, PET‐CT, and MRI showed several suspicious lymph nodes in the Axillary Levels I–III and in the supraclavicular region on the left side (Figures 1 and 2). Surgical extirpation of the supraclavicular lymph node showed simultaneous metastases of a squamous cell carcinoma and an adenocarcinoma. Further testing confirmed mixed lymph node infiltration of breast and cervical cancer cells. Immunohistochemical staining showed an inverse image related to labeling with ER (partly > 95%, partly 0%) and GATA3, p16, and p40, consistent with a synchronous manifestation of the two previously known types of carcinoma (Figures 3 and 4).

A staging PET‐CT showed no evidence of local recurrence of the cervical cancer.

The definitive diagnosis was invasive breast cancer on the left, initially cT3 cN3c cM0, G3, ER 100%, PgR < 5*%*, Her2 negative (IHC 1+) with simultaneous lymph node recurrence of cervical cancer cT0 cN0 M1 lim, and FIGO IVB.

### 2.2. Treatment

Following the recommendation of our interdisciplinary tumor conference, the patient was treated with neoadjuvant chemotherapy. To stimulate a rapid response in the cervical cancer cells, we began treatment in reverse order with four cycles of carboplatin/Taxol (corresponding to first‐line chemotherapy for cervical cancer), followed by four cycles of EC (epirubicin and cyclophosphamide) (corresponding to first‐line chemotherapy for breast cancer).

Six months later, the patient underwent tumor‐adapted reduction mammoplasty with axillary dissection on the left side and matching reduction mammoplasty on the right.

The postchemotherapy diagnosis was ypT1mi ypN3a (11/17) cM0, RCB‐II.

Adjuvant radiotherapy included internal mammary and supraclavicular node irradiation. Antihormone therapy was started with aromatase inhibitors.

The genetic testing showed no BRCA mutation; therefore, adjuvant treatment with olaparib was not indicated.

### 2.3. Outcome

The initial follow‐up examination (PET‐CT) after completion of neoadjuvant chemotherapy showed a partial response and a complete remission of the supraclavicular and axillary lymph node metastases of the cervical cancer.

A further PET‐CT four months after surgery (10/22), however, was suspicious for liver metastases. Further investigations showed breast cancer metastases. We began palliative treatment with fulvestrant and ribociclib. Unfortunately, the patient died due to tumor progression (09/23).

## 3. Discussion

The simultaneous occurrence of different cancers or cancer metastases in the same individual is rare. Nevertheless, patients with a diagnosed cancer are at a higher risk of developing additional primary cancers, which emphasizes the need for diagnostic imaging for tumor staging and follow‐up care. However, the combination of cervical and breast cancer is atypical and has only rarely been described in the literature.

The most recent published article by Kouhen et al. describes the simultaneous co‐occurrence of cervical and triple‐negative breast cancer and highlights the importance of coordinating treatment plans without compromising the effectiveness of either cancer therapy. In that case, the patient was diagnosed with squamous cell cervical cancer with parametrial infiltration. The 18F‐FDG PET‐CT showed hypermetabolic activity in the cervix as well as in the right breast and right axillary lymph nodes. Therefore, an ultrasound‐guided biopsy of the right breast was performed, and the diagnosis of triple‐negative breast cancer was made. The cervical cancer was treated with radiation therapy, including a boost and high‐dose brachytherapy, whereas chemotherapy with paclitaxel and cisplatin was administered as neoadjuvant treatment for the breast cancer. Afterwards, the patient underwent mastectomy with ipsilateral lymph node excision, and histological examination showed complete remission. Finally, the patient received radiotherapy to the right breast, chest wall, and right supraclavicular fossa. Follow‐up MRI showed no recurrence of either cervical or breast cancer [4].

Previous studies examining second primary cancers in breast cancer patients have not described cervical carcinoma [1]. One study examining a cohort of patients with cervical carcinoma (*n* = 200) regarding the occurrence of second primary cancers found only two cases of concurrent breast cancer [5]. Another study including almost 1000 women with cervical carcinoma reported a simultaneous occurrence of breast cancer in 0.6% of cases and, therefore, recommends breast examination with mammography for cervical carcinoma patients [6].

However, due to the rarity of these cases, a correlation between the simultaneous occurrence of breast cancer and cervical cancer or their metastases cannot be conclusively assessed at this time. In the presence of two different cancers, planning the best treatment with regard to the specific tumor features is particularly important. Although research on this topic is limited, treatment should be personalized and involve a multidisciplinary management approach. The combination of different chemotherapy treatments enabled effective treatment of two different tumor types. By administering combinations of systemic therapy, radiotherapy, or surgery for synchronous cancers, it may be possible to achieve complete remission of both the primary and secondary tumors, as initially shown in the presented case.

Mortality rates for the simultaneous occurrence of cervical and breast cancer are not clearly defined due to the rarity of this condition. In general, the simultaneous occurrence of different cancers is associated with a significantly higher mortality rate compared with patients diagnosed with a single cancer [7].

## Funding

No funding was received for this manuscript.

## Disclosure

All authors have read and approved the final version of the manuscript. Dr. Pösch had full access to all of the data in this study and takes complete responsibility for the integrity of the data. Dr. Pösch affirms that this manuscript provides an honest, accurate, and transparent account of the reported case report, that no important aspects of the study have been omitted, and that any discrepancies from the originally planned study have been fully explained.

## Consent

Written informed consent was obtained from the patient for publication of this case report and accompanying images.

## Conflicts of Interest

The authors declare no conflicts of interest.

## Data Availability

No datasets were generated or analyzed for this article. All information supporting this case report is provided in the cited references.
